# Binge-Like Eating Attenuates Nisoxetine Feeding Suppression, Stress Activation, and Brain Norepinephrine Activity

**DOI:** 10.1371/journal.pone.0093610

**Published:** 2014-04-02

**Authors:** Nicholas T. Bello, Chung-Yang Yeh, Jessica L. Verpeut, Amy L. Walters

**Affiliations:** 1 Department of Animal Sciences, Rutgers, The State University of New Jersey, New Brunswick, New Jersey, United States of America; 2 Graduate Program in Endocrinology and Animal Biosciences, Rutgers, The State University of New Jersey, New Brunswick, New Jersey, United States of America; 3 New Jersey Institute for Food, Nutrition, and Health, Rutgers, The State University of New Jersey, New Brunswick, New Jersey, United States of America; Monash University, Australia

## Abstract

Stress is often associated with binge eating. A critical component of the control of stress is the central norepinephrine system. We investigated how dietary-induced binge eating alters central norepinephrine and related behaviors. Young male Sprague Dawley rats received calorie deprivation (24 h) and /or intermittent sweetened fat (vegetable shortening with sucrose; 30 min) twice a week for 10 weeks. The groups were Restrict Binge (calorie deprivation/sweetened fat), Binge (sweetened fat), Restrict (calorie deprivation), and Naive (no calorie deprivation/no sweetened fat). Dietary-induced binge eating was demonstrated by Restrict Binge and Binge, which showed an escalation in 30-min intake over time. Feeding suppression following nisoxetine (3 mg/kg; IP), a selective norepinephrine reuptake inhibitor, was not evident in Restrict Binge (Restrict Binge: 107±13, Binge: 52±9, Restrict: 80±8, Naive: 59±13% of saline injection at 1 h). In subsequent experiments with Restrict Binge and Naive, Restrict Binge had reduced corticosterone (Restrict Binge: 266±25; Naive: 494±36 ng/ml) and less feeding suppression (Restrict Binge: 81±12, Naive: 50±11% of non-restraint intake at 30 min) following restraint stress (1 h). Dietary-induced binge eating in Restrict Binge was not altered by a dorsal noradrenergic bundle lesion caused by N-(2-chloroethyl)-N-ethyl-2-bromobenzylamine (DSP4), but frontal cortex norepinephrine was positively correlated with the average 30-min intake post-lesion (0.69; p<0.01). In a separate set of animals, single-unit *in vivo* electrophysiological recording of locus coeruleus–norepinephrine neural activity demonstrated reduced sensory-evoked response as a consequence of the Restrict Binge schedule (Restrict Binge: 8.1±0.67, Naive: 11.9±1.09 Hz). These results, which suggest that a consequence of dietary-induced binge eating is to attenuate the responsiveness of the brain norepinephrine system, will further our understanding of how highly palatable foods dampen the stress neuraxis.

## Introduction

Recurrent binge eating is a common behavioral feature of clinical eating disorders, including bulimia nervosa (BN) and binge eating disorder (BED). Rather than simply overeating, binge eating is accompanied by “a sense of a loss of control” over how much and what is consumed [1]. Various factors can trigger binge eating episodes, one of the most commonly reported is interpersonal stressors [2], [3]. Several studies have supported the affect regulating model of binge eating, which proposes that individuals engage in maladaptive eating behaviors to cope with negative emotions or stressful situations experienced prior to binge eating [4], [5]. A greater understanding of the relationship between binge eating and the neural pathways involved in stress control is therefore needed to identify the sustaining physiology of binge eating.

Binge foods are often dessert-like items with the predominate macronutrients being sugars and fats [6]. Consumption of sugars and fats has notable positive effects on affect and has stress-reducing properties [7], [8], [9]. In rodents, a repeated exposure to sugar, fat, or sugar + fat has been demonstrated to blunt the hypothalamic-pituitary-adrenal (HPA) axis response to stress [10], [11], [12]. Another component of the stress neuraxis is the brain norepinephrine (NE) pathways. Coordination between the NE pathways and HPA axis is needed to elicit an appropriate response to emotional stressors [13]. Alterations in brain NE are involved in the pathology of stress and affective disorders [14], [15] as well as feeding behaviors [16], [17], [18]. Along these lines, clinical studies have suggested that central NE is reduced in BN [19], [20]. Reboxetine and atomoxetine, which selectively inhibit NE reuptake, effectively reduce binge eating in BN and BED subjects [21], [22], [23]. The central NE systems emanate from two distinct neuronal populations in the brainstem, the dorsal and ventral noradrenergic bundles. The dorsal noradrenergic bundle projects mainly from the pontine locus coeruleus (LC; A6) and is the principal source of NE in the cortex, hippocampus, and cerebellum [24]. The dorsal noradrenergic bundle has been strongly implicated in attentional states, emotion, sleep, and adaptive aspects of stress reactivity [25], [26]. Because of the lack of any substantial direct dorsal bundle projections to the hypothalamus the role of the NE systems in feeding-related behavior has been largely attributed to the ventral noradrenergic bundle [17], [27]. The ventral noradrenergic bundle, which emanates from medullary regions (predominantly A1and A2) and directly projects to feeding-related hypothalamic nuclei, bed nucleus of the stria terminalis, and nucleus accumbens, has been suggested to be involved in feeding-related, reward-guided, and stress-reactive behaviors [17], [27], [28], [29]. Despite the emerging data on the ventral noradrenergic pathway, there have been few investigations into how central noradrenergic pathways are altered by dietary conditions and no specific investigations into how the dorsal noradrenergic pathway is altered by dietary conditions that promote binge eating.

In the present studies, we utilized a dietary-induced binge eating rat model to determine the consequences of binge-like eating on the central noradrenergic systems and related behaviors. This dietary-induced binge eating model incorporates repeated cycles of intermittent acute standard chow deprivation (restrict) followed by brief periods of access (30 min) to a highly palatable sugar and fat mixture (binge). Using a similar dietary-induced binge eating model, we have previously demonstrated increased neural activation of the hindbrain nucleus of the solitary tract (NTS), reduced NTS mu-opioid receptor mRNA expression, and reduced cannabinoid receptor (CB1) binding in the nucleus accumbens in rats exposed to a restrict binge feeding schedule [30], [31], [32]. In order to determine the consequences of binge-like eating on the NE systems for the present experiments, we examined the impact of the feeding suppression of nisoxetine (3-[2-methoxyphenoxy]-N-methyl-3-phenyl-1-propanamine), a selective NE reuptake inhibitor with similar affinity and selectivity for the NE transporter as reboxetine [33], in animals exposed to different variables of the restrict binge schedule. In addition, the effects of the restrict binge schedule on stress reactivity and anxiety were examined using the restraint stress-induced feeding suppression test and novelty-induced feeding suppression test, respectively. Using the neurotoxin N-(2-chloroethyl)-N-ethyl-2-bromobenzylamine (DSP4), which preferentially lesions the terminal regions of the dorsal noradrenergic bundle [34], we also examined whether this pathway was critical for the binge-like feeding behavior in animals on the restrict binge feeding schedule. Further, single-unit electrophysiology was employed to determine whether the neural response of LC neurons were altered by dietary-induced binge eating caused by the restrict binge feeding schedule.

## Materials and Methods

### Animals

Adult male Sprague Dawley rats (7–8 weeks of age) acquired from Harlan Laboratories (Frederick, MD) were individually housed and placed on a 12/12 h light- dark schedule (lights off at 1700 h). Rats were fed standard chow (Purina Rat Diet 5012l; 13% fat, 27% protein, 3.1 Kcal/g), unless otherwise noted, and water was available at all times during the experiments. All procedures were approved by the Institutional Animal Care and Use Committee of Rutgers University and were in accordance with NIH guidelines.

### Feeding schedules and experimental groups

The binge-like food used in these experiments was “sweetened fat” (8.6 Kcal/g), which consisted of vegetable shortening (Crisco, The J.M. Smucker Company, Orrville, OH) and 10% sucrose. All animals received a 24 h pre-exposure to the sweetened fat 7 days before beginning their respective feeding protocols. The pre-exposure was not only used to determine if the rats had initial differences in their preference to the sweetened fat, but also to reduce the avoidance typically associated with a novel food. The rats were divided into four groups that were designated as Restrict Binge, Binge, Restrict, and Naive. Each group initially had similar mean body weights and sweetened fat preferences. The *Restrict Binge* group (n = 9) had repeated cycles of intermittent 24 h calorie deprivation (beginning 1 h prior to lights off) followed by refeeding with standard chow and an optional 30-min access to sweetened fat. The exposure to intermittent calorie deprivation occurred on days 2 and 5, while the refeeding with standard chow and the 30-min access to the sweetened fat on days 3 and 6 of the 7-day feeding schedule. In this fashion, the Restrict Binge group was exposed to a repeated cycle that consisted of three no restriction days (days 1, 4, and 7), two weekly episodes of calorie restriction (days 2 and 5), and two weekly episodes of scheduled refeeding starting with 30-min access to an optional highly palatable food (days 3 and 6). The second group, the *Binge* group (n = 9), had ad libitum standard chow in addition to the 30-min access to the sweetened fat (days 3 and 6) at the same time and frequency as the Restrict Binge group [35], [36]. A third group, *Restrict* group (n = 8), had an identical pattern of calorie deprivation with standard chow (days 2 and 5) as the Restrict Binge group, but did not have repeated access to the optional sweetened fat upon refeeding on days 3 and 6. A *Naive* group (n = 8) had ad libitum standard chow with no access to the sweetened fat. These feeding protocols are modified from a previously published procedure for a rat model of dietary-induced binge eating [30], [31], [32]. Cumulative calorie intakes and body weights were measured once a week at the end of day 1 of the feeding schedules. Food intakes and body weights were measured to the nearest 0.1 g.

### Persistence of the overeating response following the feeding schedules and sensitivity to nisoxetine-induced feeding suppression

In order to determine the consequences of the feeding-schedule history on deprivation-induced hyperphagia, the feeding response of each group to a 24-h calorie deprivation was studied as follows. After completion of 10 weeks of their respective 7-day feeding protocol, all groups were given only ad libitum standard chow for 3 days and then deprived of food for 24 h (beginning 5 h prior to lights off). All groups were then given ad libitum access to standard chow for 24 h, and intakes were measured at 0.5 h, 1 h, 4 h, and 24 h during this refeeding period. Next, these same animals were put back on their respective 7-day feeding schedule (for 1 week only), followed by 3 days of only ad libitum standard chow and a 24-h food-deprivation period. Prior to a 24-h refeeding period, half of the rats in each of the four groups were injected with saline or nisoxetine hydrochloride (Tocris Bioscience, Bristol, UK; 3 mg/kg; IP) in order to determine noradrenergic alterations in the feeding response. This dose of nisoxetine was selected based on our previous findings that nisoxetine at 3 mg/kg differentially suppresses standard chow intake in male Sprague Dawley rats with exposure to different dietary conditions [37]. Following the saline or nisoxetine injection, all groups were given ad libitum access to standard chow for 24 h, and intakes were measured at 0.5 h, 1 h, 4 h, and 24 h. This 12-day sequence (i.e., 7-day feeding schedule, 3 days of ad libitum standard chow, 24 h of food deprivation, injection, and 24 h of refeeding) was repeated with the counterbalanced condition, in which the animals previously receiving nisoxetine were given a saline injection and those previously given saline were injected with 3 mg/kg nisoxetine.

### Feeding suppression and corticosterone response to restraint stress

A separate group of adult male Sprague Dawley rats were divided into two groups: Restrict Binge (n = 13) and Naive (n = 13). The groups were exposed to their respective feeding schedules for 10 weeks. After completion of the 10 week feeding schedule, the groups were taken off the feeding protocol for 3 days and given only ad libitum standard chow. All rats were food deprived for 24 h (beginning 5 h prior to lights off). Half of the rats from each group received a single 1 h exposure to restraint stress. The restraint stress was immobilization by placement into a well-ventilated, plexiglass flat-bottom restrainer (Plas-Labs Inc., Lansing, MI). The other half of the rats received no restraint. Following the restraint or no restraint the animals were refed ad libitum standard chow for 24 h. Intakes were measured at 0.5 h, 1 h, 4 h, and 24 h. Animals were returned to their respective feeding schedules and the counterbalanced condition was performed 7 days later. Animals were only exposed to the restraint stress once, but they were food deprived twice (a week apart). Prior to (baseline) and at the completion of the 1 h restraint stress,only when animals were exposed to the restraint stress, approximately 50 μl of blood was collected from a tail nick. The blood sample was maintained on ice until centrifugation at 3,000 rpm for 10 min at 4°C, and the plasma was stored at −80°C. A standard radioimmunoassay kit were used to determine plasma corticosterone (sensitivity: 25 ng/ml; MP Biomedicals, Santa Ana, CA) levels.

### Novelty-induced feeding suppression in two feeding groups and following nisoxetine (3 mg/kg)

Following completion of the restraint stress experiment, animals were returned to their respective feeding schedules. Two weeks later, all animals were exposed to a novelty-induced feeding suppression test, modified from Bambico and colleagues [38]. The procedure involved food-depriving all rats for 48 h (beginning 5 h prior to lights off), water was available ad libitum. All rats were refed standard chow under two conditions, in one condition half of the rats from each group were placed in a novel environment, in the other condition half of the rats were refed in their home cage. The novel environment involved moving animals into another room where they were placed in a clear acrylic crate (61×41×38 cm) with the floor covered entirely with a rough corrugated cardboard layer. On the cardboard layer were drawn 12 circles (7 cm) evenly spaced in a half-circle formation; inside each circle was one standard lab chow pellet. Each animal was placed in the clear acrylic crate in the area farthest from the chow pellet formation. Animal were placed in the novel environment for 10 min. After which time, the cardboard layer was removed with the remaining chow pellets, the area was wiped clean with a laboratory animal disinfectant (chlorine dioxide; MB-10), and a new cardboard layer along with new chow pellets was installed for the next animal. All animal behavior in the novel environment was recorded using a digital camcorder. Latency to feed, approach behavior, and time spent eating were analyzed using a time-sampling computer program, Hindsight (version 1.3). In the home environment, latency to feed was measured with a handheld timer by an observer blinded to the experimental groups. Animals were returned to their respective feeding schedule and the counterbalanced environmental condition was performed 7 days later. In a separate group of naive male rats fed only standard chow, the effect of nisoxetine (3 mg/kg; n = 6) or saline (n = 6) was examined in the novelty-induced feeding suppression. For this experiment, the procedures were identical to the novelty-induced feeding suppression protocol described above, except that animals were injected with nisoxetine or saline 1 h prior to being placed in the novel or home environment. The counterbalanced environmental condition was performed 7 days later.

### Dorsal noradrenergic bundle lesions with DSP4

A separate group of adult male Sprague Dawley rats were fed ad libitum standard chow for 3 weeks and then placed on the restrict binge feeding schedule, as described above, for an additional 3 weeks (i.e., Pretreatment). Following this, rats were injected (IP) with either N-(2-chloroethyl)-N-ethyl-2-bromobenzylamine (DSP4; 50 mg/kg; n = 6) or saline (Sham; n = 7). Because DSP4 has been demonstrated to have a minor effect on serotonin nerve terminals [34], all rats were injected with selective serotonin reuptake inhibitor paroxetine (10 mg/kg; IP) 30 min prior to the injection of DSP4 or saline [28]. Based on the recovery time with DSP4 [28], [34], 10 days after DSP4 or saline, animals were placed back on the restrict binge feeding schedule. Food intakes and body weights were measured to the nearest 0.1 g. After 5.5 weeks following injection, the rats were terminated at the expected time of the 30-min refeeding on day 3 of the 7-day feeding schedule (i.e., Post treatment). All animals were decapitated in a separate room to minimize stress. Brain tissue was immediately sectioned into 1 mm blocks using a stainless steel brain matrix (Zivic Instruments, Pittsburgh, PA) for the frontal cortex (+3.20 to +2.20 mm Bregma), medial hypothalamus (−2.12 to −3.14 mm Bregma), and cerebellum (−10.04 to −11.30 mm Bregma) regions based on anatomical markers [39]. Tissue was weighed and stored at −80°C until assayed. Individual brain sections were analyzed by reverse phase high performance liquid chromatography (HPLC; Dionex Ultimate 3000) with electrochemical detection (Coulochem III). An acetonitrile-based phosphate buffer mobile phase (Thermo Fisher Scientific, Sunnyvale, CA) was used for all experiments. The internal standard, 3,4-Dihydroxybenzylamine (DHBA), was added to all samples prior to extraction. Each brain region was individually homogenized in 550 μl of 0.1 N perchloric acid solution. Homogenates were then centrifuged at 12,000 rpm for 5 min. The supernatant was filtered using a 0.2 μm sterile nylon disposable syringe filter prior to being analyzed. This extraction procedure permitted measurements of NE, DA, and serotonin (5-HT; 5-hydroxytryptamine) by HPLC. Peak analysis was performed with Chromeleon 7.1 software (Thermofisher, Sunnyvale, CA). Monoamines were expressed as ng divided by the wet tissue weight (mg) of each sample.

### In vivo electrophysiological recording of locus coeruleus (LC) neurons

Two separate groups of animals Restrict Binge (n = 5) and Naive (n = 6), were fed on their respective feeding protocols for 10 weeks. Three days following the completion of the feeding protocols, animals were food deprived (24 h) before undergoing the procedures of *in vivo* electrophysiology. Isoflurane anesthesia was utilized to induce and maintain the animals for the duration of the recording procedures. Electrodes were fabricated from glass micropipettes and filled with 0.5 M sodium acetate and 2% pontamine sky blue (impedance 4–7 MΩ). LC single-unit spontaneous activity was recorded for at least 3 min, followed by a recording of LC sensory-evoked activity during a trial of the contralateral sciatic nerve stimulation (50 stimuli, 3.0 mA, 0.5 ms duration, 0.2 Hz). After recording spontaneous and sensory-evoked LC discharge rates, the electrode was moved ventrally (50–150 μm) until another cell was isolated, and the entire protocol was repeated. In some animals, the contralateral LC was also recorded using the described procedure. A total of 1 to 6 neuron recordings were collected per subject. Recording sites were marked by microiontophoresis of pontamine sky blue (15 μA for 10 min). The brains were subsequently removed and 40 μm frozen sections were stained with neutral red for localization of the recording site. Data were recorded with the Spike 2 software (Version 7.06; Cambridge Electronic Design) and recordings were only analyzed from those neurons that were histologically identified as being within the LC. For the peri-stimulus time histograms (PISH), synchronizing pulses initiated 2 s sweeps beginning 500 ms prior to the stimulus and the cumulative number of spikes in each 8 ms bin (250 bins total) was plotted. Thus discharge activity was recorded for 500 ms before and 1.5 s after the sciatic nerve stimulation. The 500 ms prior to each stimulus represented tonic or unstimulated discharge. Evoked discharge was defined as that which occurred after the stimulus and exceeded the mean tonic discharge rate by 2 standard deviations [40].

### Statistical Analysis

Total calorie intakes for 30 min, cumulative intakes and body weights for the 10 week feeding schedules were analyzed using a repeated measures analysis of variance (ANOVA) with feeding groups as the between-subject factor and weeks as the within subject factor. Separate repeated measures ANOVA were performed to determine the contribution of sweetened fat or standard chow on the 10-week intakes. One-way ANOVA with repeated measures was used to determine the effects of environment on the latency to feed, while one-way ANOVA was used to determine approach behavior and time spent eating. Hormone and brain catecholamine assays were analyzed with a one-way ANOVA. Post-hoc comparisons were made when appropriate with Neuman-Keuls test, unless otherwise noted. Correlation coefficients and t-test of slope  = 0 were used across groups to determine the relationship between binge intake and monoamine levels for each region. An independent t-test was used to determine a difference in LC neuronal activity, while a one-way ANOVA with repeated measures was used to analyze the PISH data. All statistical analyses were performed with Statistica 7.1 software (StatSoft Inc.) and significance was set at α = 0.05.

## Results

### Calorie intakes and body weights during 10-week feeding protocols

**Figure 1:**
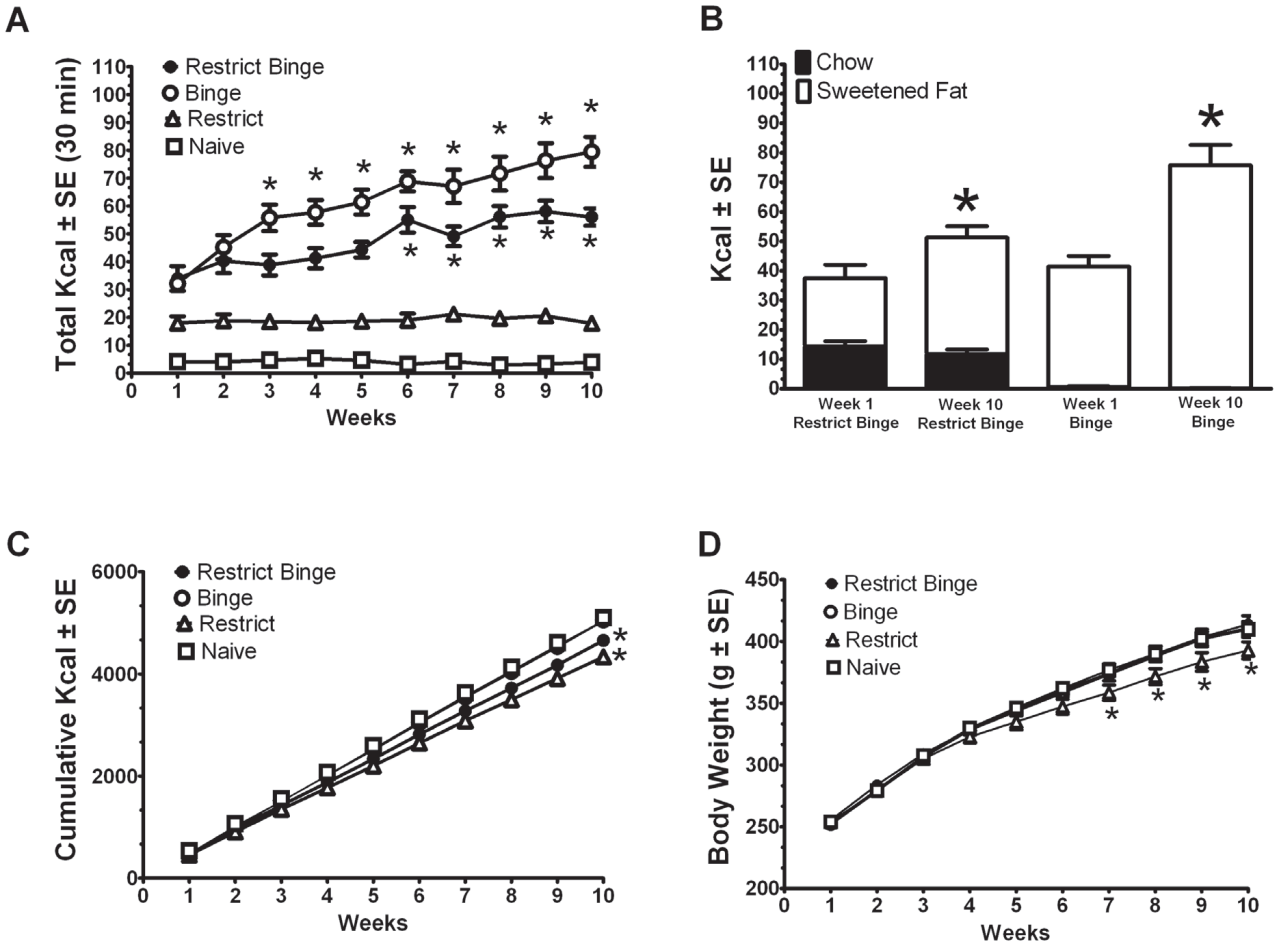
Calorie intakes and body weights during 10 weeks of the 7-day feeding protocols. Intakes (Kcal) and body weights (g) are mean ± SE. **A**: Averaged total calorie intake during the 30-min feeding session (days 3 and 6 of the feeding schedule). Restrict Binge (n = 9) and Binge groups (n = 9) had 30-min access to the sweetened fat (vegetable shortening with 10% sucrose) and standard chow, while the Restrict (n = 8) and Naive (n = 8) had access to standard chow only. Both Restrict Binge and Restrict groups had a 24 h chow deprivation prior to this 30-min access period. * indicates significance (p<0.05) from week 1 for each individual group. **B**: Calories derived from either the sweetened fat (white) or standard chow (black) during the 30-min feeding session for the Restrict Binge and Binge groups. * indicates significance (p<0.05) from week 1 for each group. **C**: Cumulative calorie intake over the 10 weeks for each group. * indicates significance (p<0.05) from the Naive group. Binge and Naive groups overlap in intake. **D**: Body weights for each group over the 10 weeks. Restrict Binge, Binge, and Naive groups overlap in body weight. * indicates significance (p<0.05) from all other groups.

In the four groups (Restrict Binge, Binge, Restrict, and Naive) we measured their food intake over time. For the 30-min total calorie intake on days 3 and 6, there was an overall group effect [F (3, 30) = 110.8, p<0.00001], time effect over the 10 weeks [F (9, 270) = 21.8, p<0.001], and group X time effect [F (27, 270) = 8.9, p<0.0001]. All four groups were different from each other (p<0.005). Only the Restrict Binge [F (9, 72) = 10.9, p<0.00001] and Binge [F (9, 72) = 15.9, p<0.0001] groups displayed an increase in calories consumed at 30-min over the 10 weeks; see Figure 1A. For the Binge group there was an increase in calories consumed from week 1 starting at week 3 (p<0.05), while the Restrict Binge group had an increase in calories consumed from week 1 starting at week 6 (p<0.05); see Figure 1A. As demonstrated in Figure 1B, this increase in calories during the 30-min period was due to an increase in the calories derived from sweetened fat (p<0.05 for both). For the cumulative calorie intake over the 10 weeks, there was a group effect [F (3, 30) = 15.7, p<0.005], time effect [F (9, 270) = 12296, p<0.0001] and group X time effect [F (27, 270) = 18.1, p<0.0001]. As demonstrated in Figure 1C, the Binge Restrict and Restrict groups consumed less total cumulative calories over time than the Binge and Naive groups (p<0.05). The body weights over the 10 weeks also showed a time effect [F (9, 270) = 1923.2, p<0.0001], and group X time effect [F (27, 270) = 2.7, p<0.001]. The Restrict group, however, was the only group that weighed less than the Naïve group (p<0.05) and this difference was evident at weeks 7, 8, 9, and 10 (p<0.05), see Figure 1D.

### Deprivation-induced hyperphagia and nisoxetine-induced feeding suppression

**Figure 2:**
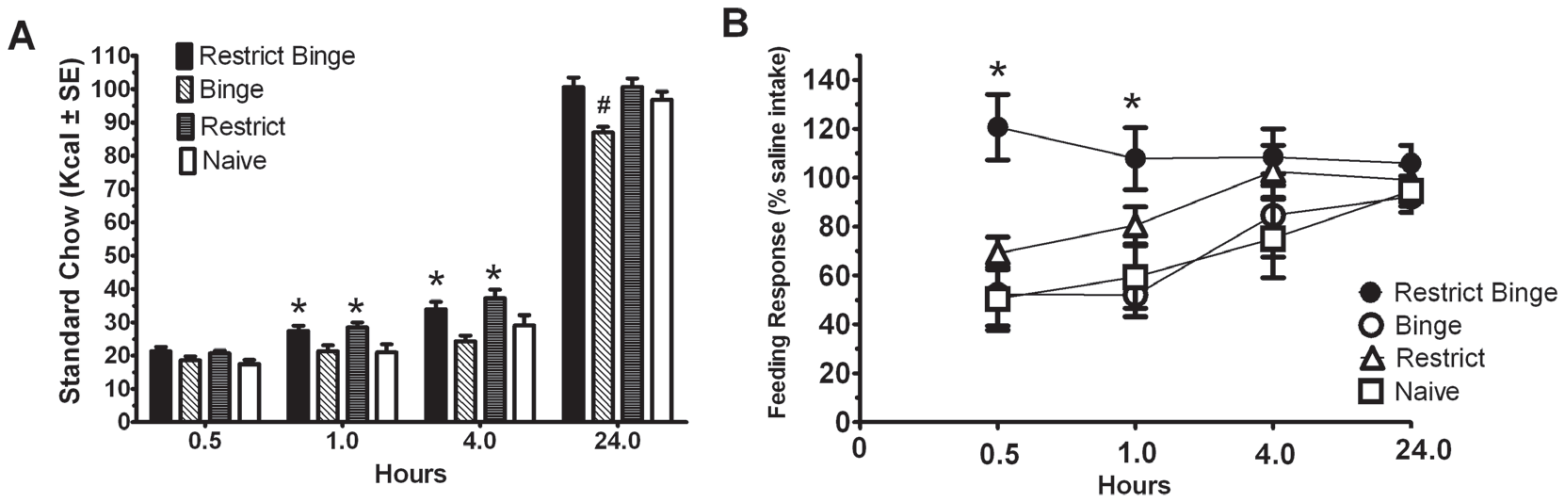
Standard chow intake in each group following 10 weeks of the 7-day feeding protocols. Intakes were measured at 0.5, 1, 4 and 24**A**: Deprivation-induced hyperphagia: Food deprivation was performed 3 days following the completion of 10 weeks of the respective 7-day feeding protocol. * indicates significance (p<0.05) from Naive group at respective time point; # indicates significance (p<0.05) from all other groups at respective time point. **B**: After a week washout, the deprivation-induced hyperphagia was repeated after an injection (IP) of nisoxetine (3 mg/kg) or saline. One week later the counterbalanced injection was performed. Similar to A, there was difference in the standard chow intake following the saline injection among groups. Data were expressed as percentage of calorie intake following the saline injection. * indicates significance (p<0.05) from all other groups.

The effects of feeding schedule history were determined by using deprivation-induced hyperphagia and measuring the feeding suppression to nisoxetine. There was an overall effect of feeding schedule history on the refeeding response [F (3, 30) = 7.5, p<0.005] and a time effect [F (3, 90) = 1717, p<0.0005]. However, dietary history X time effect approached significance [F (9, 90) = 2.0, p = 0.051]. Post-hoc tests revealed that the Restrict Binge and Restrict groups consumed more standard chow at 1 h and 4 h than Binge or Naive groups (p<0.05), whereas at 24 h the Binge group consumed less standard chow than the other groups (p<0.05), see Figure 2A. Because groups had intake differences during the saline condition identical to Figure 2A, the intake following nisoxetine administration was expressed as a percentage of the individual group's intake after saline injection. This was done to illustrate the differential response among the groups to nisoxetine. There was an overall dietary history effect [F (3, 30) = 4.6, p<0.01], time effect [F (3, 90) = 6.9, p<0.0005] and a dietary history X time effect that approached significance [F (9, 90) = 1.9, p = 0.06]. Post-hoc testing revealed an increase in the standard chow intake at 0.5 h and 1 h in the Restrict Binge group (p<0.05) compared with the other groups, see Figure 2B.

### Feeding suppression and corticosterone response to restraint stress

**Figure 3:**
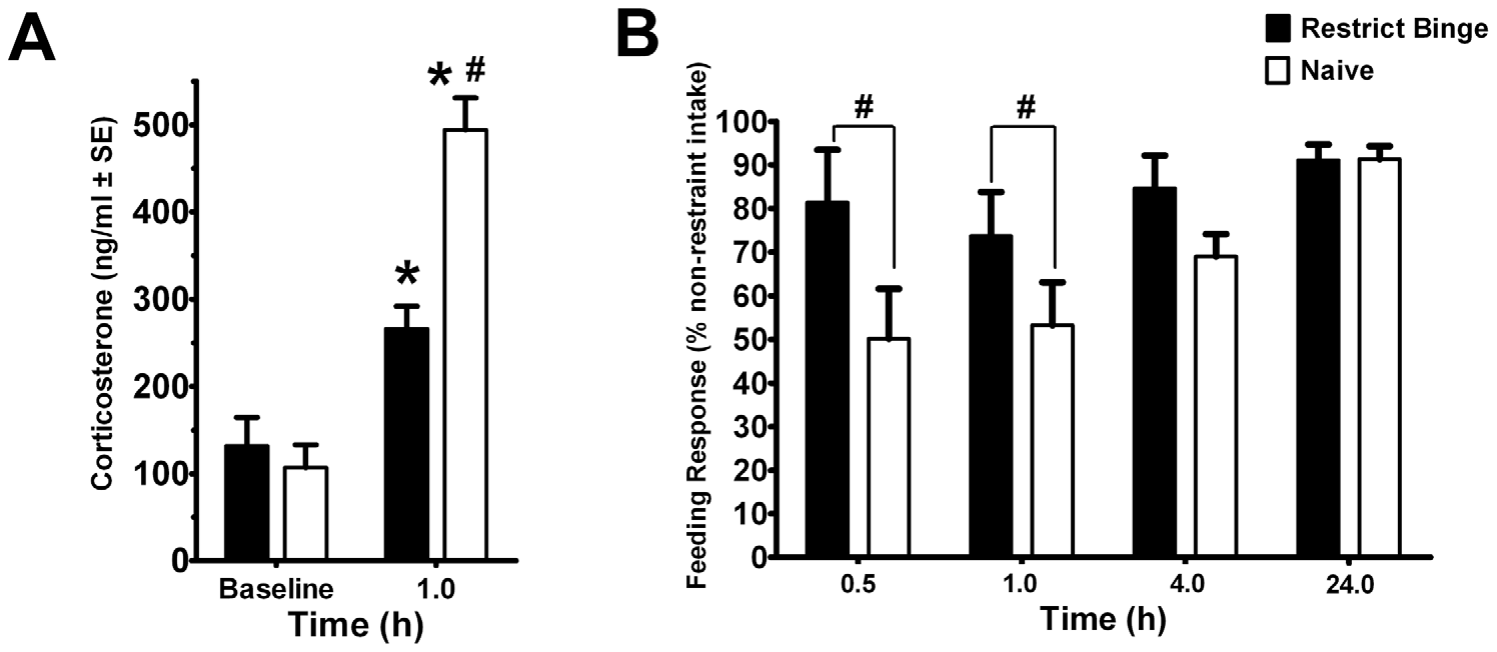
Feeding suppression and corticosterone response to restraint stress. Three days following 10 weeks of the 7-day feeding schedule of either the Restrict Binge (n = 13) or Naive (n = 13), rats underwent a 24 h food deprivation followed by an immobilization stress (restraint stress; 1 h) or no stress prior to refeeding with standard chow. One week later the counterbalanced condition was performed. **A**: Corticosterone (ng/ml) response before (Baseline) at after (1.0 h) the immobilization stressor. * indicates significance (p<0.05) from baseline levels; # indicates significant elevation (p<0.05) from Restrict Binge. **B**: Because there was a non-restraint difference in intake between groups, the data were expressed as percentage of the non-restraint intake. * indicates significance (p<0.05) between groups at the designated time point.

Because the Restrict Binge group demonstrated an overeating feeding response on standard chow and a reduced sensitivity to nisoxetine, the feeding suppression and corticosterone response to restraint stress was measured in the Binge Restrict and Naive groups. For the corticosterone response, there was an overall group effect [F (1, 24) = 9.0, p<0.01], time effect [F (1, 24) = 65.5, p<0.0005], and group X time effect [F (1, 24) = 16.9, p<0.005]. Post-hoc testing revealed that both groups had an elevation in corticosterone following the restraint stress (p<0.05), but the Restrict Binge group had reduced corticosterone at the end of the 1 h restraint period (p<0.05), see Figure 3A. Because there was a food intake difference during the non-restraint condition similar to Figure 2A, the refeeding response was normalized to the non-restraint food intake. During the refeeding response following the restraint stress, there was an overall group effect [F (1, 15) = 5.1, p<0.05], time effect [F (3, 45) = 638.5, p<0.0005] and group X time effect [F (3, 45) = 4.0, p = 0.05]. Post hoc testing revealed that the Restrict Binge group had a greater feeding response (reduced feeding suppression) at 0.5 h and 1 h (p<0.05), see Figure 3B.

### Novelty-induced feeding suppression in feeding groups and following nisoxetine (3 mg/kg)

**Figure 4:**
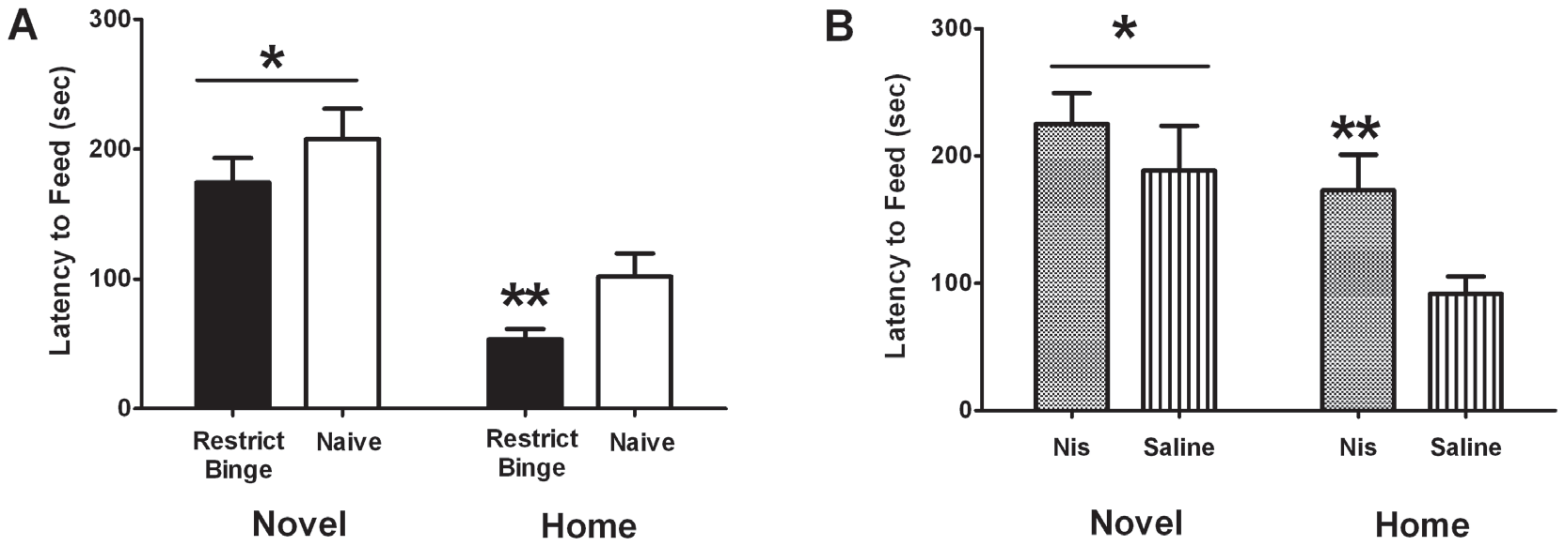
Latency to feed in the novelty-induced feeding suppression test. Rats underwent a 48**A**: Restrict Binge (n = 13) and Naive (n = 13) groups underwent the novelty-induced feeding suppression test. * indicates significance (p<0.05) from home environment; ** indicates a significant decrease (p<0.05) compared from Naive group. **B**: Separate group of naive male rats fed only standard chow were injected (IP) with nisoxetine (nis; 3 mg/kg; n = 6) or saline (n = 6) and underwent the novelty-induced feeding suppression test. * indicates significance (p<0.05) from the saline home environment; ** indicates significance from the saline home environment.

To determine how the restrict binge schedule and nisoxetine influenced anxiety-related behavior, we exposed animals to the novelty-induced feeding suppression. Feeding behavior during the novelty-induced feeding suppression test was effected by exposure to the restrict binge schedule. In the latency to feed measurement, there was a group effect [F (1, 24) = 9.8, p<0.005] and environment effect [F (1, 24) = 55.4, p<0.0001]. Post-hoc testing revealed that animals had increased latency to feed in the novel environment (p<0.05), but the Restrict Binge group had the greatest reduction in their latency to feed (i.e., fastest to feed; p<0.05), see Figure 4A. There were no group differences in approach behavior and time spent eating in the novel environment. In order to determine if the nisoxetine (3 mg/kg) dose, used in the nisoxetine-induced feeding suppression test, was anxiogenic, a separate group of rats were used to determine the effect of nisoxetine on novelty-induced feeding suppression test. There was an environment effect [F (1, 10) = 20.7, p<0.005] and nisoxetine X environment effect [F (1, 10) = 26.0, p<0.0005]. Post-hoc testing revealed that there was an increase in latency to feed in the novel environment compared with that of saline-treated animals (p<0.05), whereas injection with nisoxetine increased the latency to feed in the home environment compared with the saline-treated animals (p<0.05), see Figure 4B.

### Dorsal noradrenergic bundle lesions with DSP4

**Figure 5:**
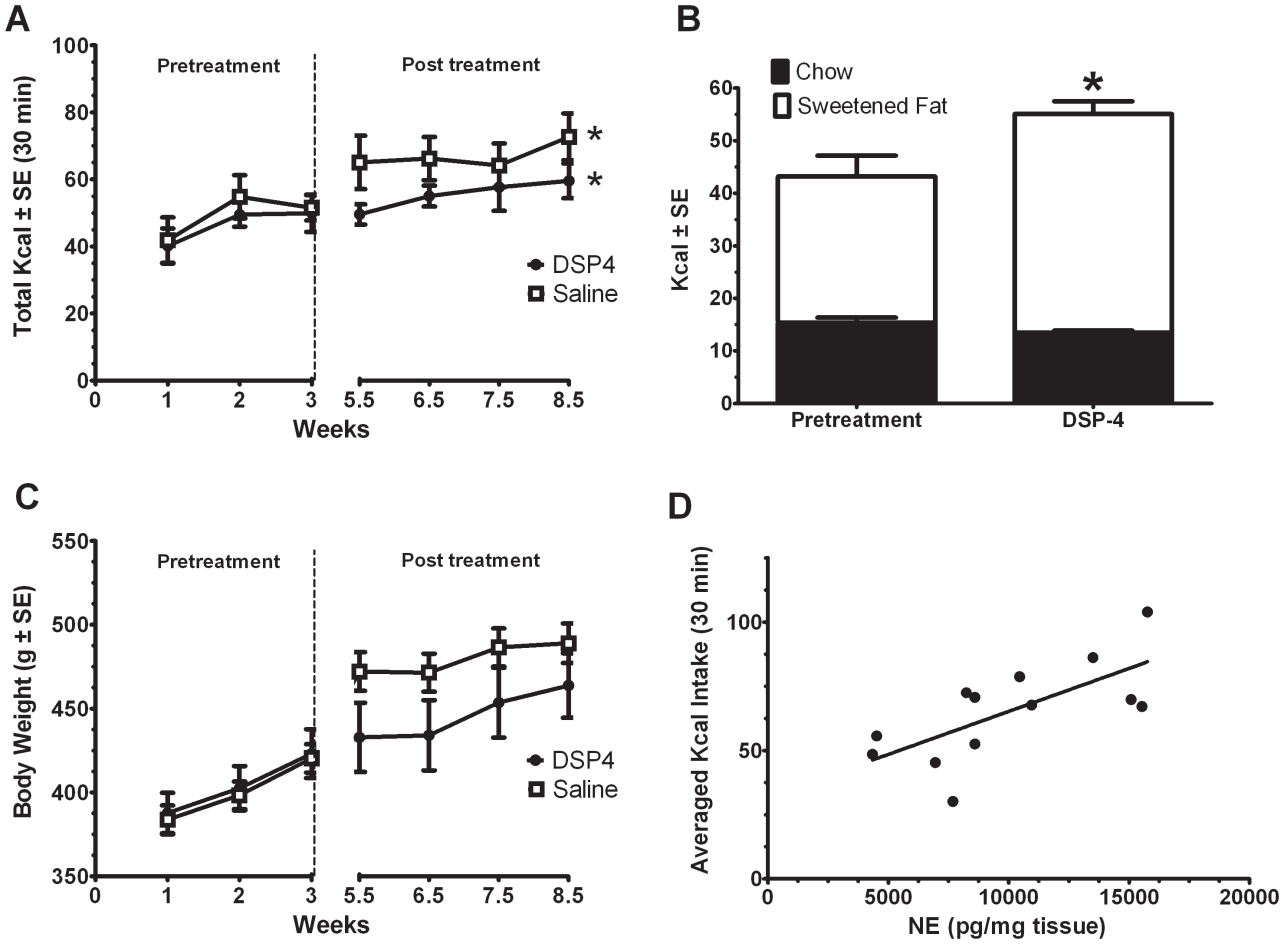
Calorie intakes and body weights following DSP4 injections in Restrict Binge. Rats were placed on the Restrict Binge feeding schedule for 3 weeks. After this time, there were injected with either DSP4 (n = 6) or Saline (n = 7). DSP4 is a selective noradrenergic neurotoxin that preferentially lesions nerve terminals of the dorsal noradrenergic bundle. **A**: Averaged total calorie intake during the 30-min feeding session (days 3 and 6 of the feeding schedule). Dotted line indicates injection of saline or DSP4, * indicates significance (p<0.05) from week 1 for each individual group. **B**: Calories derived from either the sweetened fat (white) or standard chow (black) during the 30-min refeeding session. * indicates significance (p<0.05) from pretreatment in DSP4 injected animals. **C**: Body weights for each group over the 8.5 weeks. Dotted line indicates injection of saline or DSP4. **D**: Linear fit correlation for the average calorie intake from both groups and norepinephrine content in the frontal cortex (R = 0.69; p<0.01).

In order to determine whether an intact dorsal noradrenergic system is critical for the maintenance of the binge-like eating, we injected animals with either DSP4 or saline after the binge-like eating behavior was established for 3 weeks. Following DSP4 administration, there was a separation in calorie intake between DSP4-injected and saline-injected animals, but this difference was not significant. There was, however, an increase in the 30-min Kcal intake over the 8.5 weeks [F (6, 66) = 2.93, p<0.05], with planned comparisons revealing a difference between week 1 and week 8.5 time points (p<0.05), see Figure 5A. As demonstrated in Figure 5B, for the animals injected with DSP4 the increase in calories from the pretreatment condition resulted from an increase in the calories derived from sweetened fat (p<0.05). After DSP4 or saline administration, there was an effect for the DSP4-injected animals to have a lower body weight, but this was not significantly different from the saline-treated group. There was group X weeks effect [F (3, 33) = 2.97, p<0.05], but post-hoc testing revealed the group differences only approached significance at 5.5 weeks (p = 0.08). For both groups, body weight increased over time [F (3, 33) = 68.5, p<0.005], see Figure 5C. Table 1 illustrates the monoamine content at the time of the expected refeeding for DSP4 and saline injected animals. In the frontal cortex the NE depletion approached significance [F (1, 11) = 3.58, p = 0.09], whereas there was a significant NE depletion in the cerebellum [F (1, 11) = 15.87, p<0.005] of DSP4 injected animals. With the DSP4 administration, there was no NE depletion in the hypothalamus or in the DA and 5HT content of any brain region examined. A series of correlations were performed to determine whether the amount of monoamine content in the brain regions examined was associated with averaged binge intake (i.e., total Kcal during 30-min refeeding). For this, the data was combined from the saline and DSP4 treated animals. The only region that showed a significant correlation was in the frontal cortex, which demonstrated there was a positive correlation between NE content and binge intake (0.69; p<0.01), see Figure 5D.

**Table 1:** Regional monoamine content (pg/mg tissue) of DSP-4 lesions in Dietary-Induced Binge Eating rats.

		Frontal Cortex			Hypothalamus		Cerebellum
	NE	5HT	DA	NE	5HT	DA	NE
Sham (n = 7)	11448±1229	4039±838	7528±1646	15845±3054	3659±1274	8843±2694	4623±158
Lesion (n = 6)	7727±1550	4515±683	6847±1358	15154±3106	3614±878	7994±1722	2605±567
% Depletion	33% (p = 0.09)	−18% (n.s.)	9% (n.s.)	4% (n.s.)	1% (n.s.)	10% (n.s.)	44% (p <0.005)

NE  =  Norepinephrine, 5HT = 5-Hydroxytryptamine (Serotonin), DA  =  Dopamine.

### In vivo electrophysiological recording of locus coeruleus (LC) neurons

**Figure 6:**
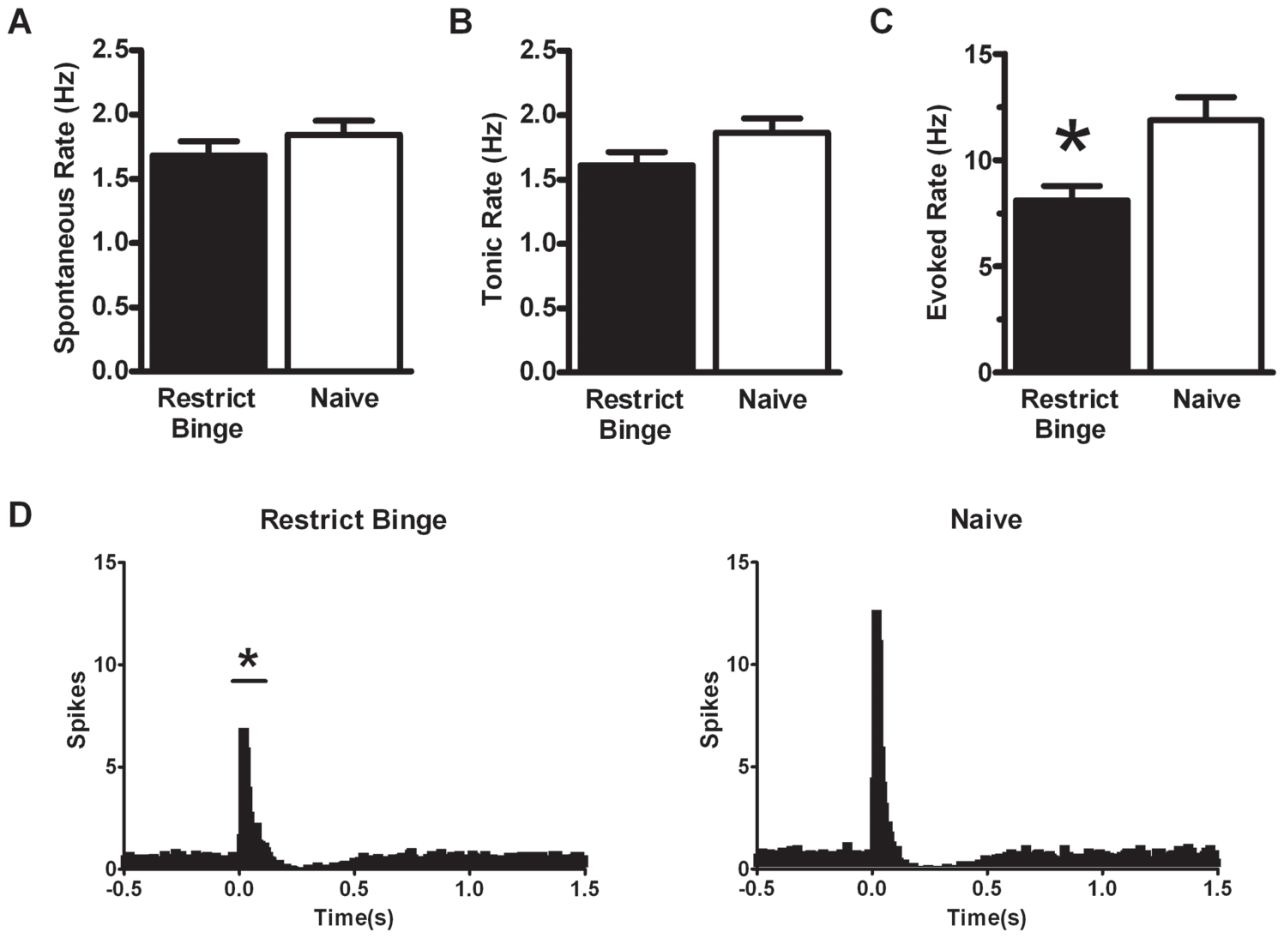
Single-unit electrophysiological recording of locus coeruleus neurons in Restrict Binge and Naive. Three days following 10 weeks of the 7-day feeding schedule of either the Restrict Binge (n = 5) or Naive (n = 6), rats underwent a 24 h food deprivation before electrophysiology. Single units from Restrict Binge (n = 26 cells) and Naive (n = 26 cells) were isolated under isoflurane anesthesia. **A**: Spontaneous discharge rate (Hz) was recorded for 3 min prior to sciatic nerve stimulation. **B**: Tonic activity was the 500 ms prior to sciatic nerve stimulation. **C**: Evoked activity was generated by sciatic nerve stimulation (50 stimuli, 3.0 mA, 0.5 ms duration, 0.2 Hz). * indicates significance (p<0.05) from Naive group. **D**: Averaged peristimulus histograms (PISH) for Restrict Binge and Naive groups. * indicates significance (p<0.05) from Naive group for evoked component (20 to 124 ms following stimulation) of the PISH.

Using single-unit electrophysiological recordings, we determined whether exposure to the restrict binge feeding protocol alters LC neural response to sciatic nerve stimulation. Data were collected using 26 neurons from the Restrict Binge group and 26 neurons from the Naive group. There was no difference in the spontaneous discharge rate between groups, see Figure 6A. Following sciatic nerve stimulations, there was no change in tonic rate, but there was a reduced sensory-evoked rate in the Restrict Binge group compared with the Naive group (t = −2.93, p<0.005), see Figures 6B and 6C. In the excitatory component (20 to 124 ms following stimulation) of the PISH, there was a group effect [F (1, 50) = 4.7, p<0.05], time effect [F (13, 650) = 33.5, p<0.0001] and group X time [F (13, 650) = 4.3, p<0.00001] with the Restrict Binge group having an overall reduced excitatory component compared with the Naive group (p<0.05). There were no significant differences in the post-stimulus inhibitory component (156 to 548 ms following stimulation) or tonic component (−500 to 0 ms before stimulation) of the PISH. The averaged PISH generated from all LC neurons for each group is illustrated in Figure 6D.

## Discussion

This study sought to examine whether dietary conditions that promoted binge-like eating alter NE-associated behaviors and neural activity. In particular, we used a model of binge eating whereby young adult male Sprague Dawley rats were exposed to repeated cycles of intermittent (twice a week) calorie deprivation followed by scheduled optional brief (30-min) access to a highly palatable sugary-fat food (sweetened fat). Binge-like eating was operationally defined in these experiments as an increase in the 30-min calorie intake over time (i.e., escalation of intake) [41]. The “Binge” group received a scheduled 30-min access to the sweetened fat and the “Restrict Binge” group received intermittent standard chow deprivation prior to the 30-min access to the sweetened fat, both satisfied this criterion. In these two groups, the escalation of intake over the 10-week feeding protocol was derived from an increase in the sweetened fat and not from the consumption of the available standard chow. The Restrict Binge group consumed more standard chow during the 30-min access than the Binge group because the Restrict Binge animals were calorie deprived. In addition, the “Restrict” group, which received intermittent calorie deprivation at the same frequency as Restrict Binge group, did not demonstrate an increase in standard chow during the 30-min period over time. We have previously demonstrated that when rats have unlimited access (24 h/7 days a week) to sweetened fat, they decrease their sweetened fat intake over time [31]. This suggests that the limited scheduled access of the sweetened fat promotes binge-like eating behavior, an effect that has been demonstrated by others using fat [42], [43], sweetened fat [12], [44], [45], and highly palatable chow [46], [47]. The lack of body weight differences among animals exposed to our dietary-induced binge eating protocol is consistent with similar binge-like eating models [48], [49], [50], suggesting the observed differences in intake are caused by the distinct feeding patterns or entrainment, rather than metabolic alterations caused by weight gain.

Because women have a higher prevalence and greater risk of developing an eating disorder than men [51], [52], one limitation of our present studies is that we used male rats. We have previously used female rats in the restrict binge feeding schedule, which demonstrated a similar specific escalation in sweetened fat intake over time [30], [32]. As a result of the gender disparity in the prevalence of eating disorders, our current ongoing experiments utilize young female rats. Nonetheless, the prevalence of eating disorders in young men are rising [53] and our findings have relevance for understanding the neural consequences of dietary-induced binge eating without the estrogen/progesterone influence.

In order to investigate the mediating influences of the norepinephrine transporter (NET) on feeding caused by the distinct dietary conditions, we examined the effect of nisoxetine on food intake. Nisoxetine is a highly selective norepinephrine reuptake inhibitor and is primarily used to identify NET kinetics and density [54], [55]. Although selective for the NET, nisoxetine has been shown to increase frontal cortical dopamine levels [56] due to the high density of NET in the frontal cortex and less discriminative nature of monoamine transporters [57], [58]. Our lab previously determined the dose response of nisoxetine (3–30 mg/kg) on feeding suppression of standard chow in adult male Sprague Dawley rats. From those experiments, we found that the feeding suppression of low dose nisoxetine (3 mg/kg) was enhanced as a result of 10 weeks on a high-fat diet (60% fat, 20% carbohydrates) and the associated weight gain [37]. In the present experiment, however, animals with a 10-week exposure to the restrict binge feeding schedule did not demonstrate a feeding suppression at 0.5 h and 1 h post-administration. Feeding suppression was demonstrated by the Binge, Restrict, and Naive groups, which indicates that the Restrict Binge group had a reduced sensitivity to the feeding suppression of nisoxetine. Such data support the assertion that there is a differential effect on NET-mediated mechanisms that is caused by dietary conditions that promote binge-like eating.

The refeeding and corticosterone response following an immobilization restraint stress test was measured in animals exposed to the restrict binge feeding schedule for 10 weeks, as well as weight-matched Naive group. The Restrict Binge group had a blunted corticosterone response to the 1 h restraint stress and also demonstrated a reduced feeding suppression upon refeeding of standard chow at 0.5 h and 1 h. A similar reduction in the hypothalamic-pituitary- adrenal (HPA) axis response has been reported as a consequence of other dietary-induced binge eating models [12] and repeated consumption of highly palatable food [10], [59], [60]. Blunted stress reactivity, including cortisol and autonomic reactivity, has been reported in human subjects with BN and BED [61], [62], [63], suggesting dysregulation of the neural controls of stress in binge eating. One limitation of our study was that animals prior to and during the restraint stress were calorie deprived, which would have increased corticosterone levels [64].

The anxiety response to a novel environment was measured using a novelty-induced feeding suppression test. While the Restrict Binge and Naive groups had no differences in their latency to feed in the novel environment, the Restrict Binge group had a reduced latency to feed (i.e., ate more promptly) in the home environment. These findings suggest that the restrict binge feeding schedule increases the food intake without decreasing the feeding response to anxiety-promoting conditions. To investigate how inhibition of the NET influenced performance in this behavioral test, we tested the effects of nisoxetine (3 mg/kg) on the latency to feed in the novel and home environments in a separate group of naive male rats. In the group of Naive rats it was shown that nisoxetine increased the latency to feed in the home environment only. Previous investigations using a novelty-induced feeding suppression test have demonstrated that acute administration of anxiolytic compounds (diazepam, chlordiazepoxide, propranolol) and chronic administration of antidepressant compounds (buspirone, desmethylimipramine) decreased the latency to feed in the novel environment, but not in the home cage, whereas the stimulant and anxiogenic compound, amphetamine, increased the latency to feed in both home and novel environments [65], [66]. Our finding that there was increased latency to feed in the home environment and not in the novel environment with nisoxetine suggests that the dose (3 mg/kg) administered was not anxiogenic. The observations that both nisoxetine and the restrict binge feeding schedule affected latency to feed in the home environment (albeit in opposite directions), without influencing the latency to feed in the novel environment suggests that the restrict binge feeding schedule influences the noradrenergic (or NET-mediated) controls of food intake.

To determine if the dorsal noradrenergic bundle is a necessary component in maintaining binge-like eating, animals were injected with DSP4 after 3 weeks of restrict binge schedule exposure. DSP4 is a neurotoxin that crosses the blood-brain barrier to act on NE terminals, but spares NE cell bodies [34]. Peripheral NE terminals recover after about a week, but central NE terminals, preferentially of the dorsal noradrenergic bundle (LC neurons), are depleted for up to 6 months [34], [67]. Following the DSP4 injection with a 10-day recovery period and 5.5 weeks on the restrict binge feeding schedule, we found a 33% and 44% depletion in NE in the frontal cortex and cerebellum, respectively. Despite this partial NE lesion of dorsal noradrenergic bundle, animals still demonstrated a continual escalation in their sweetened fat intake from the pretreatment period. These findings suggest that an intact dorsal noradrenergic bundle is not essential for the maintenance of binge-like eating in our rodent model of dietary-induced binge eating. We also found NE concentrations in the frontal cortex were positively correlated with the averaged 30-min binge intake post-treatment (i.e., DSP4 and saline). This finding further suggests that while not critical for the maintenance of the binge-like eating, NE levels in the frontal cortex were associated with bingeing behavior. One limitation of our findings is that we did not inject DSP4 into naive animals, therefore we are unable to determine whether the restrict binge feeding schedule promoted recovery NE or attenuated the NE depletion caused by DSP4. Another limitation of our study was that we did not directly measure dynamic NE levels in awake-behaving rats and our measurement of NE concentrations were from tissue homogenates at the completion of the experiments.

Using single-unit electrophysiology, we measured LC discharge rates in animals exposed to either the restrict binge or naive feeding conditions for 10 weeks. In order to standardize the stimulus to evoke LC activity we used sciatic nerve stimulation which resembles a noxious stimulus to increase LC neural activity [40]. Our results indicated that there was a reduced sensory-evoked rate in rats exposed to the restrict binge feeding schedule. These electrophysiology results when taken together with the lack of nisoxetine-induced feeding suppression, suggest that a consequence of dietary-induced binge eating is a reduction in the LC-NE pathways. This reduction in LC-mediated NE projections could also modulate the HPA axis and explain the reduced corticosterone response in the Restrict Binge group. Targeted noradrenergic immunotoxin lesions, using a saporin conjugated to dopamine beta-hydroxylase, have demonstrated that depletion of NE in the dorsal mPFC (medial prefrontal cortex) reduces corticotropin releasing factor (CRF) expression in the paraventricular nucleus of the hypothalamus (PVN) and plasma corticosterone in response to restraint stress [13]. Projections from the mPFC, which are glutamatergic, do not innervate the medial PVN directly but send projections to the bed nucleus of the stria terminalis (BNST) anterior region (fusiform and dorsal medial nuclei) [68]. The anterior BNST send GABAergic projections to the medial PVN to inhibit HPA axis activity [69], [70]. Because NE in the mPFC inhibits the descending glutamatergic pathway to the GABAergic anterior BNST [13], an increase in NE in the mPFC indirectly increase the neuroendocrine response to stressors (i.e., NE releases the “brakes” on HPA –axis activity). One likely modulating effect of the HPA axis, therefore, could be a decrease in NE in the mPFC, although NE levels were not directly measured in the present study. Future experiments will be needed to determine directly whether a decrease in NE concentrations in the mPFC is a consequence of dietary-induced binge eating.

The etiology of clinical binge eating (and associated eating disorders) is multi-factorial and comprises a complex interaction between environmental and genetic susceptibility. For these reasons, developing a clinically appropriate animal model for binge eating is difficult. As such, clinical and pre-clinical research has focused on whether dieting, dietary restraint, and/or psychological stress are precursors or risk factors to binge eating in at-risk populations [71], [72]. Our novel findings indicate that one consequence of dietary-induced binge eating is to dampen the HPA axis and reduce the neural activity of the LC-NE system. These findings further our understanding of how binge eating behavior could impact stress reactivity.
